# Does Reconstructive Surgery Improve Psychological Recovery After Burn Injury?

**DOI:** 10.7759/cureus.117120

**Published:** 2026-09-29

**Authors:** Daniel Gravino, Kyle Attard

**Affiliations:** 1 Surgery, Mater Dei Hospital, Msida, MLT; 2 Psychiatry, Mater Dei Hospital, Msida, MLT

**Keywords:** anxiety, autologous fat grafting, body image, burn injury, depression, post-traumatic growth, post-traumatic stress disorder, quality of life, reconstructive surgery, social participation

## Abstract

Burn reconstruction aims to restore function, comfort, and appearance, but its effect on psychological recovery remains less certain. This narrative review aimed to examine depression, anxiety, post-traumatic stress disorder (PTSD), demoralization, body image, social participation, post-traumatic growth (PTG), neurocognitive recovery, and health-related quality of life (HRQoL) after burn reconstruction. PubMed/MEDLINE was searched from inception to September 17, 2026, with citation chaining and targeted searches for reconstructive modalities and validated outcome instruments. The evidence is heterogeneous and predominantly observational. Contracture release and laser scar treatment consistently improve selected functional, symptom, and burn-specific quality-of-life outcomes. Autologous fat grafting is promising for scar quality and satisfaction, but comparative evidence remains limited. In contrast, large Burn Model System analyses do not demonstrate consistent improvements in broad mental-health scores among survivors undergoing later surgery; these adjusted associations identify ongoing vulnerability but do not establish that reconstruction causes psychological harm. Depression, anxiety, PTSD, demoralization, body-image distress, and cognitive impairment should therefore be measured as distinct outcomes rather than inferred from scar appearance or generic HRQoL. The most defensible model is one of integrated care: reconstructive surgery combined with rehabilitation, trauma-informed communication, realistic expectation setting, psychological screening, active follow-up, and timely mental-health referral. Future studies should use procedure-specific preoperative baselines, repeated assessments, appropriate comparators, Burn Care Quality Platform (BCQP) variables, and validated burn-specific participation and scar-impact measures.

## Introduction and background

Modern burn care has improved survival, which has shifted attention toward long-term function, appearance, participation, and emotional health. Survivors may live with pain, pruritus, contracture, altered facial expression, visible scarring, sleep disturbance, unemployment, and difficulty resuming social or intimate roles [1-3]. Psychological morbidity is common: depression, anxiety, and post-traumatic stress symptoms may persist long after wound closure, and body-image disturbance and reduced quality of life can remain clinically important even when the burn is medically stable [4-7].

For patients, psychological distress is often linked to concrete losses and fears. Depression may accompany the loss of appearance, function, independence, employment, relationships, or a previous sense of identity. Anxiety may center on pain, the prospect of another operation, the return of contracture, public scrutiny, fear of negative evaluation, or shame about visible difference. Positive adaptation can also occur; post-traumatic growth (PTG) describes changes such as greater appreciation of life, altered priorities, personal strength, or stronger relationships, and it can coexist with persistent distress [8]. Demoralization is another relevant construct. It includes helplessness, hopelessness, loss of meaning, and a perceived inability to cope. It overlaps with, but is not synonymous with, major depression. A 2024 cross-sectional burn study found substantial demoralization and linked it to social support, hope, coping, socioeconomic factors, and burn characteristics [9].

Burn injury can also affect neurological and cognitive recovery. Reviews describe central nervous system effects associated with inflammation, blood-brain barrier disruption, sensory and motor disturbance, delirium, and longer-term problems with memory, attention, and executive function [10,11]. These deficits can influence consent, rehabilitation adherence, return to work, and responses to patient-reported outcome measures, and they may coexist with depression, anxiety, or post-traumatic stress disorder (PTSD). Psychological assessment after burns should therefore avoid assuming that every difficulty with concentration, motivation, or participation is purely psychiatric.

Post-burn reconstruction includes contracture release, grafting, local or free flaps, tissue expansion, scar revision, laser treatment, and autologous fat grafting. These procedures can restore movement, eyelid or oral competence, wound stability, contour, and appearance. They can also introduce pain, donor-site morbidity, rehabilitation demands, additional scars, repeated anaesthesia, and disappointment when the final appearance does not match expectations. Therefore, achieving the intended anatomical or functional surgical result cannot be assumed to guarantee psychiatric recovery.

The central evidence gap is not whether reconstruction can improve scars or function; many studies show that it can. The unresolved question is whether those physical gains translate into measurable improvement in depression, anxiety, PTSD, demoralization, body image, social participation, PTG, and broader health-related quality of life (HRQoL), and which outcomes are most sensitive to reconstructive benefit. This review synthesizes the available evidence, explains why studies appear to disagree, and proposes a practical framework for integrating psychological and neurocognitive assessment into reconstructive burn care.

## Review

Methods and scope

A structured narrative search of PubMed/MEDLINE was conducted from database inception to September 17, 2026. Search concepts included combinations of burn or burn scar with reconstruction, reconstructive surgery, scar revision, contracture release, plastic surgery, fractional carbon dioxide laser, autologous fat grafting, lipofilling, depression, anxiety, PTSD, post-traumatic stress, PTG, body image, psychosocial outcome, patient-reported outcome, quality of life, social participation, Life Impact Burn Recovery Evaluation (LIBRE), Brisbane Burn Scar Impact Profile (BBSIP), Burn Care Quality Platform (BCQP), long-term outcome, expectations, and propensity score. Backward citation searching was conducted from eligible primary studies and major reviews. English-language human studies were prioritized if they assessed at least one prespecified outcome after post-acute burn reconstruction. Studies of acute wound closure alone, cosmetic treatment of non-burn scars, and technical reports without patient-reported outcomes were excluded from the central synthesis. General burn-psychology reviews, registry guidance, and measurement papers were retained to provide context and to aid interpretation of outcome instruments.

Because the literature consists of dissimilar procedures, populations, follow-up periods, and measures, statistical pooling was not appropriate. Evidence was synthesized by reconstructive modality and psychological domain. The review was designed as a narrative review rather than a systematic review; it did not include duplicate independent screening, protocol registration, formal risk-of-bias scoring, or a PRISMA flow diagram. Accordingly, the absence of an identified study should not be interpreted as proof that no study exists, and conclusions are framed around direction and consistency rather than pooled effect size.

Why psychological recovery may follow reconstruction

Reconstruction can influence psychological health through several linked pathways. Functional correction may improve self-care, mobility, speech, oral competence, eyelid closure, hand use, sexual function, and capacity to work. Relief of chronic pain, pruritus, and heat sensitivity may improve sleep and reduce irritability. Improvement in visible scars or asymmetry may reduce appearance-related self-consciousness and avoidance. These changes can increase social participation and self-efficacy, which may in turn reduce depressive symptoms. HRQoL reviews consistently show that physical function, pain, social participation, and mental health are interdependent after burns [11].

The same pathway contains several failure points. Anatomical correction may be incomplete, contractures can recur, scars may remain conspicuous, and repeated procedures can extend the patient's identity as a surgical patient [12]. Expectations may exceed the biological limits of reconstruction. Depression can reduce rehabilitation engagement, while PTSD-related avoidance may make examinations, dressings, anaesthesia, or hospital environments difficult. Financial strain and time away from work may offset functional benefit. Psychological response is therefore likely to depend on whether the chosen procedure addresses the patient's personally important problem, not simply whether a scar score improves.

A deficit-only model is also incomplete. Burn survivors can report PTG, including greater appreciation of life, changed priorities, stronger relationships, personal strength, or spiritual development, while still experiencing depression, anxiety, or PTSD. A 2024 systematic review found generally moderate PTG across burn cohorts but substantial heterogeneity in measurement and associated factors [13]. Reconstruction may create conditions that support positive adaptation by restoring agency, role participation, or valued function; no current evidence demonstrates that an operation itself causes PTG. Assessment should therefore use a dual-axis model that records both distress and positive adaptation without making growth an expectation or implying that persistent distress represents failure.

Direct clinical evidence

Direct evidence is limited and heterogeneous (Table 1). The largest studies come from the United States Burn Model System. Sheckter et al. analyzed 1,359 adult survivors from five centres; 372 underwent at least one reconstructive operation within 24 months. After propensity matching, scar operations performed more than six months after injury were associated with improved physical-component scores, but mental-component scores were unchanged or lower after scar and contracture procedures [14-15]. The persistence of these findings after measured adjustment is clinically meaningful and should not be reduced to unadjusted injury severity. It indicates that survivors requiring reconstruction remain a psychologically vulnerable group even when physical outcomes improve. Causal direction nevertheless remains unresolved because procedure-specific preoperative symptoms, time-varying indication, scar visibility, patient priorities, and rehabilitation exposure were not fully represented.

**Table 1:** Key studies linking post-burn reconstruction with psychological or quality-of-life outcomes Studies are summarized as reported in the cited publications; observational associations do not establish a causal effect of surgery. Range-of-motion percentages are reproduced from the source and should be interpreted using its functional reference definition. Observational associations should not be interpreted as proof of a causal psychological effect of surgery BBSIP: Brisbane Burn Scar Impact Profile; BSHS-B: Burn Specific Health Scale–Brief; CO₂: carbon dioxide; EQ-5D: EuroQol five-dimension instrument; PERMA: positive emotion, engagement, relationships, meaning, and accomplishment; POSAS: Patient and Observer Scar Assessment Scale; SF-36: 36-Item Short Form Health Survey

Study	Design and sample	Intervention or setting	Relevant findings	Main limitation
Thombs et al., 2007 [16]	Cross-sectional; 224 adults seeking burn reconstruction	Reconstructive consultation	High prevalence of depressive symptoms reported; body-image dissatisfaction was strongly associated with depressive symptoms	No postoperative assessment; association does not establish treatment effect
Sinha et al., 2016 [17]	Prospective pre-post cohort; 31 enrolled, 24 followed at 3 months	Reconstructive surgery in a mission setting	Significant improvement in SF-36 physical and mental component scores observed; work impairment decreased	Small sample, attrition, short follow-up, and no comparator
Issler-Fisher et al., 2017 [18]	Prospective cohort; 47 patients and 118 scars	One ablative fractional CO2 laser treatment	Scar thickness, texture, color, symptoms, and BSHS-B quality of life improved over short follow-up	Uncontrolled; repeated scar-level observations and short-term assessment
Sheckter et al., 2020 [14]	Prospective multicentre database; 1,359 survivors, 372 with reconstruction	Scar, contracture, or open-wound surgery within 24 months	After propensity matching, physical scores improved after later scar surgery; mental-component scores were unchanged or lower	Adjusted association is important, but causal direction remains unresolved; global mental scores may miss appearance-specific change
Issler-Fisher et al., 2021 [19]	Prospective case-control; 167 laser-treated and 20 controls	Single ablative fractional CO2 laser treatment	Scar thickness, pain, pruritus, POSAS patient scores, and BSHS-B improved; largest domain differences included affect and body image	Nonrandomized groups and small control cohort
Hendriks et al., 2021 [1]	Pre-post cohort; 44 patients and 115 joints; 86% follow-up at 12 months	Contracture release in Tanzania	Functional range of motion rose from 37.3% to 108.7%; disability-free survival from 55% to 97%; EQ-5D utility from 0.69 to 0.93	No psychiatric measures or control group
Balaji et al., 2022 [20]	Small observational study; 30 patients	Surgery for post-burn contracture deformity	Depression was common; improvement in disability was associated with improvement in depressive symptoms	Conference-supplement abstract, unclear design details, and no comparator
Liu et al., 2022 [21]	Nested case-control study in patients with extensive burn scars	Ablative fractional CO2 laser compared with conventional scar surgery	Reported improvement in general and burn-specific quality of life after laser treatment	Observational comparison; psychiatric diagnoses were not primary outcomes
Luna et al., 2023 [15]	National longitudinal database; 727 survivors, 201 with later surgery	Scar- or contracture-related surgery after discharge	Negative anxiety, depression, and fatigue associations persisted after adjustment for severity and available confounders	No procedure-specific baseline; time-varying indication and unmeasured patient priorities limit causal interpretation
Buta et al., 2024 [22,23]	Prospective cohort; 113 adults with hypertrophic burn scars	Reconstruction, skin grafting, and/or laser treatment	Evaluated adult BBSIP reliability, factor structure, and longitudinal score change in a reconstructive cohort	Conference-supplement reports; limited causal inference and longer-term comparative data
El Sewify et al., 2025 [3]	Systematic review; 14 studies and 885 patients	Autologous fat grafting for burn scars	Studies generally reported improved scar quality, function, or satisfaction; pooled inference was not possible	One randomized trial and predominantly uncontrolled case series; direct psychiatric outcomes were uncommon
Wu and Zhong, 2026 [24]	Retrospective cohort; 102 patients undergoing scar plastic surgery	PERMA-based psychological nursing plus usual care versus usual care	Adjunct intervention was associated with better anxiety, depression, post-traumatic stress symptoms, and quality of life	Tests an added psychosocial program, not surgery alone; retrospective design

A subsequent Burn Model System analysis of 727 survivors, including 201 who underwent scar- or contracture-related surgery after acute hospitalization, found worse PROMIS anxiety, depression, and fatigue scores among operated survivors after adjustment for burn severity and available confounders [15]. The adjusted association is therefore a population-level signal, not a crude comparison. It still does not establish that surgery causes psychological harm: database timing did not define a procedure-specific baseline or isolate symptom change around one operation, and unmeasured clinical need, visibility, expectations, access, and staged treatment may influence both surgery and outcome. Together, the two multicentre studies support a conservative conclusion: reconstruction may improve selected physical outcomes, but current comparative data do not demonstrate reliable improvement in broad mental-health scores and justify integrated mental-health care throughout reconstruction.

Smaller studies are more encouraging but less resistant to bias. At reconstructive consultation, Thombs et al. found that 46% of 224 patients scored at least 10 on the Beck Depression Inventory; body-image dissatisfaction was strongly associated with depressive symptom severity, while physical function added relatively little explanatory value [16]. This cross-sectional study did not test surgical benefit, but it identifies the population entering reconstruction as psychologically vulnerable and suggests that appearance-related distress deserves direct measurement. In a short prospective cohort from a surgical mission setting, Sinha et al. reported improvement in SF-36 physical and mental component scores and the Work and Productivity Impairment measure three months after reconstruction; only 24 of 31 enrolled patients completed follow-up, and there was no comparator [17]. A Tanzanian cohort of 44 patients undergoing release of 115 contracted joints showed large and sustained gains in range of motion, disability-free survival, and EQ-5D utility at 12 months [1]. These results support functional and global quality-of-life benefit but do not establish change in depression, anxiety, or PTSD.

Evidence by reconstructive Modality

Contracture Release and Functional Reconstruction

Contracture release offers the most direct functional mechanism for psychological benefit [1]. Restoration of movement can reduce dependence, enable return to work, and improve participation. The Sinha and Hendriks cohorts demonstrate that meaningful gains in physical function and global quality of life are achievable [1,17]. The multicentre evidence nevertheless shows that global mental-component scores may not move in parallel [14]. One explanation is that surgery corrects a discrete functional bottleneck but leaves trauma symptoms, social stigma, chronic pain, and socioeconomic disruption largely unchanged. Another is measurement: a broad mental-component score may be less responsive than an appearance-specific or participation-based instrument.

Small observational data suggest that improvement in disability can accompany improvement in depressive symptoms [20], but temporal association alone cannot separate the effects of surgery, rehabilitation, attention from the clinical team, and natural recovery. Recurrence and the need for postoperative splinting and therapy are also critical. A technically adequate release without access to rehabilitation may not produce sustained functional or psychological benefit.

Scar Revision and Soft-Tissue Reconstruction

Excision, grafting, local rearrangement, flaps, and tissue expansion are used for unstable scars, contour deformity, exposed structures, and visible disfigurement. Direct psychiatric outcome data for these heterogeneous procedures are sparse. The Burn Model System analyses combine procedures into broad categories and therefore cannot determine whether facial, hand, breast, perineal, or lower-limb reconstruction has a distinct psychological effect [14,15]. This matters because the value of a change depends on visibility, function, identity, and the patient's goals. A small improvement in eyelid closure or oral competence may be life-changing despite minimal change in a generic scale, whereas a cosmetically noticeable but functionally minor change may matter most through body image.

Autologous Fat Grafting

Autologous fat grafting is increasingly used as a regenerative adjunct for adherent, painful, dyschromic, or poorly pliable burn scars and contour defects [25]. A 2025 systematic review identified 14 studies involving 885 patients and found broadly favorable reports for scar quality, pliability, function, satisfaction, and patient-reported outcomes [3]. Across three studies using the Patient and Observer Scar Assessment Scale (POSAS), patient scores improved, though standardized reporting was limited [3]. The review's apparent consistency should therefore be interpreted in light of a literature dominated by case series and subjective outcomes.

Randomized evidence remains limited. A small prospective randomized double-blind split-scar pilot study in eight paediatric burn survivors found no appearance advantage for fat grafting over saline at six to 12 months [26]. Lipofilling should consequently be represented in reconstructive outcome research but not portrayed as an established psychological treatment. Future trials should distinguish volume restoration from scar regeneration, standardize processing and injection technique, record repeat sessions and donor-site burden, and pair objective pliability and POSAS outcomes with BBSIP, body-image, LIBRE participation, and mental-health measures.

Laser Scar Treatment

Ablative fractional carbon dioxide laser has the most consistent patient-reported evidence within contemporary scar reconstruction [2]. Prospective cohort and case-control studies report improvements in scar thickness, pain, pruritus, patient scar ratings, and Burn Specific Health Scale-Brief (BSHS-B) quality of life [18,19]. In the larger case-control study, between-group differences were greatest in affect, body image, heat sensitivity, treatment burden, and work [19]. A nested case-control study in survivors with extensive scars also reported improvement in general and burn-specific quality of life after laser therapy [21]. Systematic review evidence supports improvement in scar characteristics, although protocols and outcome measures remain heterogeneous [27].

These findings are clinically relevant but should not be overextended. BSHS-B affect and body-image domains are not equivalent to a psychiatric diagnosis, and most laser studies were not designed to test depression, anxiety, or PTSD. Symptom relief may improve mood indirectly, but repeated procedures, anaesthesia, cost, and access can influence overall benefit. Randomized comparisons with standardized mental-health outcomes remain uncommon.

Outcomes by psychological domain

Depression

Depressive symptoms after burns may reflect pain, disability, sleep disturbance, unemployment, social withdrawal, pre-existing vulnerability, and dissatisfaction with appearance. The high prevalence of depressive symptoms among patients seeking reconstruction and the strong association with body-image dissatisfaction indicate that surgical clinics are an important point for detection [16]. However, evidence that reconstruction itself treats depression is mixed. Small before-and-after studies suggest improvement in mental-component or depressive symptom scores [17], while larger comparative datasets show unchanged or worse depression-related outcomes in operated groups [14,15]. The balance of evidence therefore supports screening and integrated treatment, not an assurance that mood will improve once the scar is revised.

Anxiety

Anxiety may concern procedures, pain, recurrence, public scrutiny, work, or the uncertainty of final appearance. Direct before-and-after anxiety studies after burn reconstruction are scarce. The Burn Model System analysis found greater anxiety symptom burden in survivors who underwent later surgery [15], despite adjustment for measured burn severity; residual confounding by ongoing treatment need remains possible. A 2026 retrospective study reported better anxiety outcomes when a nursing model based on positive emotion, engagement, relationships, meaning, and accomplishment (PERMA) was added to perioperative scar surgery care [24]. Although this does not isolate a surgical effect, it suggests that perioperative psychological intervention may modify distress that surgery alone does not reliably address.

Post-traumatic Stress Disorder

PTSD is linked to the injury event, pain, frightening intensive-care experiences, perceived threat to life, repeated procedures, and reminders of the burn. Contemporary meta-analysis suggests that approximately one in five burn survivors may experience clinically significant PTSD, with prevalence generally declining over time but persisting in a subgroup [8]. Reconstruction does not directly process traumatic memories or avoidance, and operative care may expose patients to sensory and environmental triggers. Despite frequent discussion of PTSD in burn rehabilitation, direct studies testing change in validated PTSD outcomes before and after reconstruction are notably lacking. The available PERMA-based cohort suggests that structured psychosocial care alongside surgery may reduce post-traumatic stress symptoms [24], but prospective controlled confirmation is needed.

Post-traumatic Growth and Positive Adaptation

Psychological recovery after burns is not limited to symptom remission. PTG describes positive changes that can emerge through the struggle with trauma, including greater appreciation of life, personal strength, changed priorities, enhanced relationships, or spiritual development. A 2024 systematic review found that burn survivors generally reported moderate PTG, although estimates and explanatory factors varied across settings and instruments [13]. PTG can coexist with PTSD, depression, grief, disability, or dissatisfaction; it should not be used to minimize distress or to prescribe a preferred emotional response.

No identified study establishes that burn reconstruction causes PTG. A plausible pathway is indirect: restored hand use, facial function, comfort, or participation may increase agency and reopen valued roles, creating opportunities for meaning-making. Conversely, repeated surgery can extend uncertainty and treatment burden. Reconstructive studies should therefore measure PTG prospectively alongside symptoms and participation, ideally using the Post-Traumatic Growth Inventory or a validated short form [13], while avoiding cross-sectional claims that higher growth scores represent cure.

Body Image and Social Reintegration

Body image is the domain most intuitively linked to reconstruction, yet objective appearance and subjective adjustment are not interchangeable. A systematic review found mixed associations between burn injury or scar characteristics and body image, reflecting heterogeneous measures and predominantly cross-sectional designs [10]. Longitudinal work suggests that body-image dissatisfaction can mediate the relationship between burn severity and psychosocial adjustment [28]. Perceived stigmatization and fear of negative evaluation represent distinct pathways to body-image dissatisfaction and self-esteem [29]. Earlier work likewise found that body-image dissatisfaction relates to social and functional outcomes after burns [30].

Reconstruction may improve contour, color, texture, symmetry, and functional appearance, but patient-perceived benefit depends on scar salience, cultural meaning, prior experiences of staring or unwanted questions, and agreement between the patient's goals and the achievable result. Appearance change can support social reintegration by reducing camouflage behavior, avoidance of photographs or public spaces, and anxiety during intimate, educational, or occupational encounters. It can also disappoint when residual scarring remains conspicuous or when surgery creates a new donor-site scar. Social recovery should therefore be measured directly through participation, relationship, return-to-work or education, and stigma-related outcomes rather than inferred from clinician-rated scar improvement [31].

The laser literature provides direct evidence of improvement in BSHS-B body-image and affect domains [19]. Appearance-specific assessment is increasingly established, particularly through the BBSIP. The BBSIP was developed from burn-survivor input as a burn-scar-specific health-related quality-of-life measure and has demonstrated longitudinal validity, responsiveness, and interpretability in adults [32-40]. In 2024, two prospective reports evaluated the adult BBSIP in 113 patients with hypertrophic burn scars undergoing reconstruction and/or laser treatment, including its reliability, factor structure, and score change over time [22,23]. These reconstructive-cohort data make the BBSIP particularly relevant when the intervention targets visible or symptomatic scarring.

The BBSIP complements rather than replaces other instruments. POSAS links patient and observer scar ratings; SCAR-Q measures appearance, symptoms, and psychosocial impact across scar types; the Satisfaction With Appearance Scale (SWAP) focuses on burn-related appearance satisfaction; and BSHS-B places body image within broader burn-specific recovery [40-45]. A reconstructive study should pair a measure such as BBSIP, SCAR-Q, or SWAP with depression, anxiety, and PTSD scales because improvement in appearance-related distress is not equivalent to remission of a psychiatric disorder [33-47. Results should be reported by subscale and anchored to patient goals, anatomical site, visibility, and social participation.

Social reintegration should be measured with a burn-specific participation instrument rather than a generic quality-of-life proxy. The LIBRE Profile was developed in 601 burn survivors and provides computer-adaptive or fixed short-form assessment across family and friends, social interactions, social activities, work and employment, romantic relationships, and sexual relationships, with reported scale reliability of 0.84-0.93 [46-54]. It is proposed here as a suitable burn-specific participation instrument for reconstructive cohorts [54]. Pairing LIBRE with BBSIP allows investigators to test whether change in scar impact translates into resumption of social roles rather than assuming reintegration from appearance or clinician-rated scar change alone.

Quality of Life

HRQoL shows the most consistent postoperative improvement, especially when the intervention relieves a contracture or symptomatic scar. Contracture release cohorts demonstrate substantial gains in mobility, disability, and utility [1], while laser cohorts show improvement in scar-specific quality of life [21]. Yet HRQoL is a multidimensional endpoint. Improved physical function, work, or pain may raise a total score even when depression or PTSD remains unchanged. Conversely, a generic mental-component score may remain stable while appearance confidence improves. Reports should therefore present domain-level results rather than treating a single total score as proof of psychological recovery. Table 2 summarizes the direction, limitations, and narrative confidence assessment of the evidence for each outcome domain.

**Table 2:** Overall interpretation of evidence by outcome domain Author-developed narrative synthesis based on the studies cited in each row [1,7,13-15,17,19,23,54]. Confidence categories are qualitative judgements about the consistency and limitations of the evidence, not the results of a formal evidence-grading assessment. Confidence ratings represent narrative judgements based on study design, consistency, comparators, outcome measurement, and risk of confounding; they are not formal GRADE ratings BBSIP: Brisbane Burn Scar Impact Profile; HRQoL: health-related quality of life; LIBRE: Life Impact Burn Recovery Evaluation; PTG: post-traumatic growth; PTSD: post-traumatic stress disorder

Domain	Direction of evidence	Confidence	Interpretation
Depression	Mixed	Low	Small pre-post studies suggest benefit; larger observational cohorts do not demonstrate reliable improvement and may show greater burden in operated survivors [34-35]
Anxiety	Insufficient direct evidence	Very low	Few studies measure perioperative change. Greater anxiety in operated cohorts persisted after measured adjustment, but causal direction remains unresolved [35]
PTSD	Insufficient direct evidence	Very low	Reconstruction does not directly target trauma memories; validated longitudinal PTSD assessment is rarely reported [36]
PTG	Unknown reconstructive effect	Very low	Moderate PTG is reported in burn cohorts, but no study establishes that reconstruction causes positive adaptation [37]
Body image and participation	Probable domain-specific benefit in selected patients	Low	Laser and BBSIP data support scar-specific change; LIBRE should test whether this translates into social-role recovery [23], [38]
HRQoL	Generally favourable for function and scar symptoms	Low to moderate	The most consistent gains occur in mobility, disability, pain, pruritus, work, and burn-specific quality of life; mental subscales remain mixed [4]

Why the studies disagree

The apparently conflicting literature should not be interpreted simply as positive versus negative studies. First, confounding by indication is a major concern. Patients selected for further surgery have residual scars, deformity, pain, contracture, instability, or functional impairment severe enough to justify intervention. They may therefore have greater baseline psychological burden than patients who do not undergo reconstruction. Importantly, unfavorable psychological associations in Burn Model System studies persisted after adjustment for measured burn severity and available confounders [10,11]. These findings should therefore not be dismissed as crude severity effects. However, adjustment cannot account for variables that were never measured, including procedure-specific preoperative distress, scar visibility, expectations, evolving indication, coping, patient priorities, and rehabilitation exposure.

Second, uncontrolled pre-post studies may overestimate benefit through natural recovery, regression to the mean, concurrent rehabilitation, increased clinical attention, and changing expectations. Third, timing varies substantially. Early reconstructive intervention may occur while spontaneous recovery continues, whereas late surgery may target chronic or treatment-resistant problems. Fourth, reconstructive procedures differ fundamentally. A hand contracture release, facial flap, skin graft, laser procedure, and fat grafting treatment have different biological, functional, aesthetic, and psychological objectives.

Fifth, follow-up is important. Patients who return repeatedly to reconstructive clinics may differ systematically from those who disengage from care. Finally, outcome selection shapes the interpretation. A generic mental-component score may fail to identify improvement in scar symptoms, confidence, appearance satisfaction, or social participation. Conversely, improvement in a scar-specific instrument cannot establish remission of major depression or PTSD.

Measurement and study design

A minimum outcome set should separate psychiatric symptoms, positive adaptation, body image, scar symptoms, function, participation, and HRQoL. Screening and assessment guidance for burn survivors supports staged use of validated instruments rather than informal clinical impression [48]. Routine psychosocial screening and implementation of patient-reported outcome measures within burn outpatient services are feasible and may improve longitudinal recognition of psychological and functional needs [55-59]. Selection of HRQoL instruments should also consider their measurement properties within burn populations. PROMIS-29 has undergone validation in adult burn survivors and may provide a useful generic multidomain measure alongside burn-specific instruments.

Validated screening tools are preferable to informal clinical impressions [26]. The Burn Specific Health Scale-Brief (BSHS-B) measures burn-specific domains including affect, body image, work, physical function, and treatment burden [27]. The Patient and Observer Scar Assessment Scale (POSAS) permits parallel patient and clinician evaluation of scar characteristics [28].

SCAR-Q evaluates appearance, symptoms, and psychosocial impact across scar populations [29,30], while the Satisfaction With Appearance Scale (SWAP) was developed specifically to assess appearance concerns among burn-injured patients [31].

The Brisbane Burn Scar Impact Profile (BBSIP) provides a burn-scar-specific survivor-derived assessment and has demonstrated psychometric validity in adults with burn scars [32]. More recent prospective reports have evaluated its psychometric properties and longitudinal score changes among adults undergoing reconstruction, grafting, and/or laser treatment, making it particularly relevant to reconstructive cohorts [33,34].

Social reintegration should be measured directly. The Life Impact Burn Recovery Evaluation (LIBRE) Profile assesses participation across family and friendships, social interactions, social activities, employment, romantic relationships, and sexual relationships [35].

In 2024, the American Burn Association incorporated long-term outcome variables into the full Burn Care Quality Platform (BCQP) for outpatient follow-up after hospital discharge. These include follow-up, wound closure, post-discharge procedures, scar assessment, pain, pruritus, and documentation of selected psychological conditions [36,37]. Early implementation demonstrated both the potential of this framework and incomplete capture of some long-term variables [36]. The BCQP framework provides an opportunity to harmonize national data collection while additional validated patient-reported measures address psychological, scar-specific, and participation outcomes. This is summarized in Table 3.

**Table 3:** Standardized outcome framework aligned with BCQP long-term outcomes The author-proposed framework informed by published psychological screening guidance [48], instrument development and evaluation studies [1,17,22,23,32,49-65], the PTG review [13], BCQP reports [55,56], and patient-priority research [65,66]. Suggested instruments and follow-up intervals are recommendations for prospective reconstructive research; the complete framework has not been validated and is not presented as an official BCQP requirement. Registry fields and timing must follow the applicable BCQP data dictionary BBSIP: Brisbane Burn Scar Impact Profile; BCQP: Burn Care Quality Platform; BSHS-B: Burn Specific Health Scale–Brief; EQ-5D: EuroQol five-dimension instrument; GAD-7: 7-item Generalized Anxiety Disorder scale; HRQoL: health-related quality of life; IES-R: Impact of Event Scale–Revised; LIBRE: Life Impact Burn Recovery Evaluation; LTO: long-term outcome; PCL-5: Posttraumatic Stress Disorder Checklist for the Diagnostic and Statistical Manual of Mental Disorders, Fifth Edition; PHQ-9: 9-item Patient Health Questionnaire; POSAS: Patient and Observer Scar Assessment Scale; PROMIS: Patient-Reported Outcomes Measurement Information System; PROMIS-29: 29-item PROMIS profile; PTG: post-traumatic growth; PTSD: post-traumatic stress disorder; SCAR-Q: the named patient-reported outcome instrument assessing scar appearance, symptoms, and psychosocial impact; SF-12: 12-Item Short Form Health Survey; SF-36: 36-Item Short Form Health Survey; SWAP: Satisfaction With Appearance Scale

Domain	Suggested measure	Timing	Purpose
BCQP LTO core	Current BCQP data-dictionary fields: follow-up status, wound closure, post-discharge procedures, scar assessment, pain, pruritus, and documented depression, post-traumatic stress disorder, and acute stress disorder [55,56]	At each registry-defined outpatient follow-up; retain exact field definitions and version	Enable national benchmarking and linkage while avoiding incompatible local recoding
Depression	PHQ-9 or another validated depression scale [48]	Consultation; preoperative baseline; 3, 6, and 12 months	Detect clinically important symptoms and measure their trajectory independently of quality of life
Anxiety	GAD-7 or PROMIS Anxiety [48]	Consultation; preoperative baseline; early postoperative and longer-term follow-up	Separate general anxiety from procedure-related distress
Post-traumatic stress	PCL-5 or IES-R [48]	Baseline and 6- and 12-month follow-up; earlier if symptoms are evident	Identify persistent trauma symptoms and possible procedure-related triggering
Positive adaptation	Post-Traumatic Growth Inventory or a validated short form [13]	Preoperative baseline; 6 and 12 months	Measure positive adaptation separately from symptom reduction without implying that post-traumatic growth is obligatory
Burn-scar impact	Adult BBSIP; report item groups and subscales [22,23,32]	Preoperative baseline; 3, 6, and 12 months; before each staged procedure	Capture burn-scar symptoms, appearance, emotional reactions, relationships, social interaction, activity, and treatment burden
Appearance and psychosocial impact	SWAP or the SCAR-Q psychosocial and appearance scales [51-53]	Baseline; 3, 6, and 12 months	Capture appearance-specific outcomes and permit triangulation with BBSIP findings
Scar symptoms and severity	POSAS with pain and pruritus scales [50]	Baseline and at each treatment follow-up	Relate subjective and clinician-assessed scar changes to psychological outcomes
Burn-specific HRQoL	BSHS-B [49]	Baseline; 3, 6, and 12 months	Measure affect, body image, work, physical function, treatment burden, and other burn-specific domains
Social participation	LIBRE Profile with return-to-work or return-to-education status [54]	Preoperative baseline; 6 and 12 months	Measure family, social, vocational, romantic, and sexual role trajectories directly
Generic HRQoL	PROMIS-29, SF-12, SF-36, or EQ-5D [1,17,60-65]	Baseline; 6 and 12 months	Support comparison across conditions and health-economic analyses
Patient goals and expectations	Ranked functional and aesthetic goals, goal-attainment scaling, visual-aid review, and teach-back [65-68]	Consultation; preoperative review; final follow-up	Identify differences between functional and aesthetic priorities, recalibrate unrealistic expectations, and assess concordance between stated goals and achieved outcomes

Improving causal inference

Randomized comparison would provide the clearest evidence where it is ethical and practical, but many reconstructive decisions depend on anatomy, symptoms, patient preference, timing, and surgeon judgement. Observational studies should therefore define the treatment eligibility date, index procedure, preoperative baseline, comparator, treatment-assignment window, and follow-up period before analyzing outcomes. Propensity-score matching or weighting can improve comparability between treated and untreated groups when sufficient overlap exists, but investigators should report the propensity model, covariate balance, overlap, and relevant diagnostics [61]. These approaches can address measured confounding but cannot eliminate bias from unmeasured variables. Sensitivity analyses should therefore assess the potential influence of unmeasured confounding and test the robustness of the observed associations [62]. Repeated-measures models should also account for baseline symptom score, centre effects, staged operations, rehabilitation exposure, complications, attrition, time-varying treatment, and missing data.

Clinical implications

Current evidence supports a combined reconstructive and psychological pathway rather than isolated surgical treatment. Psychological screening should begin at reconstructive consultation because distress can influence goals, consent, rehabilitation adherence, and postoperative appraisal [26]. A positive screening result should prompt appropriate clinical assessment rather than automatic exclusion from reconstructive treatment.

Functional and aesthetic expectations should be considered separately. Research examining patient expectations after burns demonstrates that patient priorities may differ from clinician-defined outcomes [42]. A surgeon may regard increased range of motion or durable wound coverage as success while the patient is primarily concerned about color, symmetry, visibility, social confidence, or return to a particular role. Patients should therefore be encouraged to identify and rank their major goals, including movement, pain or pruritus, appearance, social confidence, employment, intimacy, independence, and treatment burden. Outcomes important to burn survivors during scar management extend beyond clinician-rated scar characteristics [43].

Expectation management should be realistic but should not pathologize hope. Standardized photographs, discussion of residual scarring and donor sites, explanation of staged procedures, and teach-back may help ensure that functional and aesthetic expectations correspond to what is biologically achievable. Trauma-informed perioperative care is also important. Predictable communication, adequate analgesia, consent before examination or touch, and continuity of care may reduce avoidable distress in survivors with trauma-related symptoms.

Burn recovery requires coordinated management of pain, itch, psychological adjustment, rehabilitation, and social function [44]. Qualitative research in burn and reconstructive populations additionally suggests that emotional distress may be overlooked when clinical attention is concentrated predominantly on physical treatment [45]. A multidisciplinary pathway should therefore involve reconstructive surgery, physiotherapy and occupational therapy, scar management, pain management, psychology or psychiatry where indicated, social support, and peer support where available.

Failure to attend follow-up should not automatically be interpreted as recovery or lack of interest. Burn survivors may encounter transportation, financial, employment, psychological, or treatment-fatigue barriers. Loss to follow-up can introduce both clinical risk and research bias [38]. Where appropriate and feasible, missed appointments should prompt proportionate outreach and re-engagement, particularly where significant psychological or functional needs have previously been identified [39].

Discussion

The available evidence supports a more nuanced understanding of psychological recovery following burn reconstruction than a simple question of whether surgery improves mental health. The most consistent benefits of reconstruction occur in outcomes directly targeted by treatment: movement, disability, scar symptoms, pain, pruritus, appearance-related concerns, and burn-specific quality of life. Contracture release provides substantial functional benefit in selected patients [13,14], while fractional CO2 laser treatment has demonstrated reproducible improvement in symptomatic and patient-reported scar outcomes [15-17,21]. Autologous fat grafting is promising but remains supported primarily by uncontrolled studies, and randomized evidence remains limited [19,20,67-70].

Evidence for direct improvement in depression, anxiety, or PTSD is considerably less certain. Large Burn Model System analyses demonstrate persistent psychological burden among survivors requiring further surgery, even after adjustment for measured injury characteristics [10,11]. These findings should not be dismissed, but neither should they be interpreted as evidence that surgery itself causes psychological harm. Patients requiring reconstruction represent a selected population. They continue to experience deformity, contracture, pain, functional impairment, appearance concerns, or other sequelae severe enough to warrant additional treatment. Persistent psychological symptoms may therefore partly reflect the indication for reconstruction rather than the effect of the procedure.

Measurement also contributes to apparent disagreement between studies. Generic mental-health scores may fail to detect important improvement in body image, scar impact, comfort, function, or social participation. Conversely, improvement in a scar-specific or appearance-specific scale does not establish resolution of a psychiatric disorder. Psychological recovery is additionally influenced by factors extending beyond the scar itself. Loss of previous function or identity, fear of pain or judgement, demoralization, chronic neurological symptoms, unemployment, relationships, rehabilitation access, and expectations may all shape the patient's postoperative experience.

The appropriate clinical interpretation is therefore not that reconstruction does or does not improve psychological recovery universally. Rather, reconstruction may facilitate psychological recovery when it restores meaningful function, reduces symptoms, improves an important appearance concern, or enables return to a valued role. Whether this translates into improvement in depression, anxiety, PTSD, demoralization, participation, or PTG depends on the broader clinical and psychosocial context. This supports an integrated model in which reconstructive surgery, rehabilitation, pain and neurological management, psychological screening, expectation alignment, and social reintegration are treated as interconnected components of long-term burn care.

Future research should move away from broad comparisons between patients who did and did not undergo “reconstructive surgery” and instead evaluate defined procedures from a true preoperative baseline using procedure-specific comparators and multidimensional patient-reported outcomes. The BCQP long-term outcome infrastructure, supplemented with BBSIP, LIBRE, psychiatric measures, and patient-defined goals, offers a practical framework for achieving this.

Limitations

This narrative review has several limitations. First, PubMed/MEDLINE was the primary structured database, supplemented by targeted searches and backward citation searching. Embase, PsycINFO, Scopus, and Web of Science were not systematically searched. Relevant multidisciplinary, non-English, or unpublished evidence may therefore have been missed. Second, duplicate independent screening and formal risk-of-bias assessment were not undertaken. Evidence quality was instead assessed narratively according to study design, sample size, comparator, baseline assessment, validated outcome measurement, follow-up, attrition, and control of confounding.

Third, the underlying reconstructive literature is heterogeneous. Studies differ substantially in burn severity, anatomical site, treatment modality, timing, number of procedures, rehabilitation exposure, follow-up interval, and outcome measurement. Fourth, much of the evidence is observational. Even propensity-adjusted analyses cannot exclude residual confounding by treatment indication, scar visibility, psychological vulnerability, expectations, access to care, or rehabilitation engagement. Fifth, psychiatric outcomes are rarely the primary endpoint of reconstructive studies. Improvement in body image, scar symptoms, function, or HRQoL should therefore not automatically be interpreted as remission of depression, anxiety, or PTSD.

Sixth, evidence regarding demoralization, PTG, neurological symptoms, and social participation specifically following reconstruction remains limited. These domains are clinically relevant to recovery, but their causal relationship with reconstructive surgery has not been established. Finally, some relevant reconstructive studies are small, uncontrolled, or available only as conference reports, which limits certainty regarding treatment effects.

Research priorities

Future research should focus on the following priorities: develop prospective, procedure-specific cohorts with a clearly defined eligibility date, index reconstructive procedure, preoperative baseline, and an appropriate delayed-treatment or non-operative comparator [10,11]; align registry data with BCQP long-term outcome variables while reporting centre participation, follow-up completion, missingness, and linkage success [36,37]; collect common psychological and reconstructive outcomes, including depression, anxiety, PTSD, BBSIP, BSHS-B, LIBRE, scar symptoms, body image, patient goals, and PTG at comparable preoperative, three-, six-, and 12-month intervals [8,26-35]; use stronger methods to address confounding, including propensity-score matching or weighting, overlap and balance diagnostics, and sensitivity analyses for unmeasured confounding [40,41]; report procedure-specific outcomes rather than combining contracture release, grafting, flap reconstruction, laser therapy, and fat grafting into one heterogeneous treatment category; measure expectations prospectively, distinguishing anticipated functional improvement from aesthetic change and comparing these expectations with postoperative patient-reported outcomes [42]; assess clinically meaningful change, including responder proportions and domain-level outcomes rather than relying only on statistical significance or global summary scores; measure neurological and sensory symptoms, including neuropathic pain, weakness, sensory disturbance, and nerve-compression symptoms when relevant to the reconstructed anatomical region [9]; measure social recovery directly, including return to employment or education, relationships, participation, financial burden, and treatment burden [35,43]; study demoralization and PTG separately from depression, acknowledging that hopelessness, loss of meaning, psychiatric symptoms, and positive adaptation represent related but distinct constructs [7,8]; extend follow-up for at least 12 months where possible and distinguish the effects of surgery from concurrent rehabilitation and structured psychological interventions; and co-design outcome priorities with burn survivors so that successful reconstruction reflects function, comfort, identity, appearance, relationships, and participation rather than clinician-rated scar characteristics alone [43,46].

## Conclusions

Post-burn reconstruction can improve physical function, scar symptoms, appearance-related outcomes, participation, and burn-specific quality of life, particularly after contracture release and contemporary scar treatment. However, evidence for direct improvement in depression, anxiety, or PTSD remains limited and inconsistent. Psychological recovery after burns is influenced not only by the reconstructed scar but also by perceived loss, fear, pain, neurological symptoms, identity, expectations, relationships, social participation, rehabilitation, and the personal meaning of both the injury and its treatment. Demoralization and PTG further show that adjustment cannot be adequately represented by a single mental-health score.

Reconstructive surgery should therefore be viewed as one component of comprehensive burn recovery rather than a stand-alone psychiatric treatment. The most appropriate clinical pathway combines careful reconstructive planning with rehabilitation, validated psychological assessment, trauma-informed care, realistic expectation setting, active follow-up, and access to appropriate mental-health and social support. Future studies should use procedure-specific preoperative baselines, prospective comparators, validated multidimensional outcome measures, adequate follow-up, and stronger methods for addressing confounding. Ultimately, reconstructive success should be judged not only by the scar that clinicians can see, but also by the patient's function, comfort, agency, identity, relationships, participation, and emotional health.
